# Long-term outcomes of adjuvant proton radiotherapy (PRT) for residual pituitary adenoma (PA) in adults – a retrospective, single institute experience

**DOI:** 10.1007/s11060-026-05669-2

**Published:** 2026-06-12

**Authors:** Fabian J. K. Allmendinger, Maximilian Deng, Sebastian Regnery, Lars Wessel, Katharina Kozyra, Felix Englert, Ricarda Wickert, Jannik Walter, Lucas Mose, Thomas Tessonnier, Sandro M. Krieg, Jürgen Debus, Laila König, Tanja Adena-Eichkorn

**Affiliations:** 1 Department of Radiation Oncology, Heidelberg University Hospital, Heidelberg University, Im Neuenheimer Feld 400, 69120 Heidelberg, Germany; 2 Heidelberg Institute for Radiation Oncology (HIRO) and National Center for Radiation Research in Oncology (NCRO), Heidelberg, Germany; 3 National Center for Tumor Diseases (NCT), NCT Heidelberg, A Partnership Between DKFZ and Heidelberg University Hospital, Heidelberg, Germany; 4 Heidelberg Ion-Beam Therapy Center (HIT), Department of Radiation Oncology, Heidelberg University Hospital, Heidelberg University, Heidelberg, Germany; 5 Hopp Children’s Cancer Center Heidelberg (KiTZ), Heidelberg, Germany; 6 Clinical Cooperation Unit Radiation Oncology, German Cancer Research Center (DKFZ), Heidelberg, Germany; 7 Department of Neurosurgery, University Hospital Heidelberg, Heidelberg, Germany

**Keywords:** Pituitary adenoma, Adjuvant proton radiotherapy, Real-world evidence, Long-term follow up, Local tumor control

## Abstract

**Purpose:**

Pituitary adenomas (PA) are benign neoplasms treated by resection. Gross total resection is limited by extrasellar expansion in proximity to critical neurovascular structures. Residual tumor remains at risk of progression, with serious consequences such as optic compression or hormone hypersecretion. Adjuvant radiotherapy (RT) offers a rescue modality for both, impeding regrowth and hormonal relapse. Proton radiotherapy (PRT), owing to its favorable dose distribution and reduced exit dose through the Bragg peak, further expands the armamentarium of conventional RT modalities in cases of extrasellar macroadenomas in contact with vulnerable organs at risk. Hence, the present study provides initial evidence on the efficacy and toxicity of adjuvant PRT and offers insight into the decision-making criteria for selecting PRT over photon-based techniques.

**Methods:**

Adjuvant PRT applied for 22 residual PA including 16 non-functioning- (NFPA) and 6 functioning PA (FPA) at a large particle-therapy center, who were reviewed for local tumor control, toxicity, hormone status and visual function. Assessment of PRT response accounted for neuro-oncology criteria.

**Results:**

After a median follow up of 65 months 5-year local tumor control rate of PA (FPA and NFPA) and hormone control rate of FPA was 100%. Secondary hypopituitarism after PRT was rare (1/22). Despite treatment escalation with PRT, stable endocrine status during follow-up enabled significant reductions in required glucocorticoid supplementation. Visual improvement was reported in 64%. Toxicity was limited, with fatigue and headache being most frequent.

**Conclusion:**

Adjuvant PRT for complex residual PA is efficacious and safe for treating residual disease in extrasellar expansion.

**Importance of the study:**

Pituitary adenomas (PAs) with extrasellar extension in proximity to vulnerable neurovascular tissue or brain parenchyma carry risk of residual tumor after resection that is not amenable to further safe resection. Subsequent tumor regrowth may compress adjacent neurovascular structures resulting in frequent sequelae such as visual deterioration or central nerve palsies. Persistent hormone-secreting adenoma may further sustain endocrine dysregulation. Adjuvant radiation therapy represents an important treatment strategy to impede regrowth while minimizing toxicity to surrounding critical structures, thus achieving durable visual improvement and long-term hormonal control. Proton radiotherapy (PRT) may further improve the therapeutic ratio by exploiting its steep dose gradient and highly conformal dose distribution to enhance normal tissue sparing without compromising tumor control in selected complex PA. Therefore, the present study reports experience with adjuvant PRT for complex residual PA, demonstrating 5-year local tumor control of 100%, visual improvement in 64%, and stable hormone levels in 95%.

**Supplementary Information:**

The online version contains supplementary material available at 10.1007/s11060-026-05669-2.

## Introduction

Although benign, PAs cause substantial morbidity related to endocrine activity and local mass effects. Owing to the sellar location, macroadenomas compress adjacent neurovascular structures, particularly the optic system, cranial nerves and brain parenchyma, precipitating vision loss, headaches, and nerve palsies [1, 2]. Endocrine dysregulation, either from functioning PA (FPA) or sellar-compressing, non-functioning PA (NFPA), causes endocrine disorders including Cushing`s disease, acromegaly or hypopituitarism [3–5]. To account for this neuroendocrine origin, PA have been redefined as pituitary neuroendocrine tumors (PitNETs).

While microsurgical resection remains the cornerstone of tumor removal and sellar decompression, surgery in the extrasellar region is inherently challenging. Complete resection is often not feasible in case of cavernous macroadenomas, as these are of high risk for visual impairment and vascular injury. As a result, subtotal resections are reported in approximately 60% of patients [6, 7] all of which regrowing in up to 60% [8, 9]. This incidence necessitates further consideration of additive strategies to preserve long-term tumor, hormone and neurological control.

Available adjuvant treatment for residual PA covers active surveillance, repeated surgical resection, radiotherapy or medical therapy for FPA. Radiotherapy plays a central role in reaching secondary but durable tumor control, which is of utmost relevance for those cases not feasible for further surgery. Both conventional fractionated radiotherapy (RT) and stereotactic radiosurgery (SRS) have demonstrated excellent long-term tumor control rates exceeding 90% at 10 years [10–14].

Thereby, SRS is typically reserved for small, well-circumscribed lesions with sufficient safety margins to the optic apparatus, cranial nerves or carotid arteries. In contrast, large, irregular target volumes near organs at risk (OARs) are often unsuitable for SRS, as adequate target coverage may conflict with nearby OAR constraints and preclude delivery of an effective prescription dose. In these situations, fractionated RT remains a standard approach. However, even with modern highly conformal, image-guided photon techniques, RT delivers an unavoidable entrance and exit dose along multiple beam paths, resulting in an integral dose bath to surrounding tissues limiting sufficient target dose coverage in complex PA in contact to OARs.

Proton beam radiotherapy (PRT) offers a dosimetric advantage to improve normal-tissue sparing through an inverse depth dose profile (Bragg peak phenomenon), enabling steep dose fall-off beyond the target and reducing irradiation of adjacent OARs [15]. This may expand the available armamentarium of RT-techniques for borderline PA not sufficiently targetable by conventional RT. Although modern RT techniques have significantly lowered the incidence of late toxicity [6, 16–21], irradiation of surrounding structures may still contribute to delayed adverse effects such as hypopituitarism, optic neuropathy, radiation necrosis, seizures, or secondary malignancies.

Despite theoretical advantages, the clinical evidence supporting the use of adjuvant PRT remains limited. Criteria in the decision-making for PRT are yet not defined or discussed in the treatment of PA. The present study is the first to report a long-term institutional experience of adjuvant PRT in patients with complex residual PA, giving insight into applied criteria in the multidisciplinary decision-making process for PRT in each individual case.

## Methods

### Study design

Retrospective, single-center, observational study conducted at the department of radiation oncology with a large particle radiotherapy unit (Heidelberg ion beam therapy center). Real world data was investigated between 2013 and 2024. Study design according to Strengthening the Reporting of Observational Studies in Epidemiology guidelines [22] (Supplement 2).

### Data collection and patient characteristics

22 adult cases receiving adjuvant PRT for residual PA were identified through our institutional database by ICD-Code (D35.2) or name of disease. Clinical data was collected from the clinical information system (i.s.h.med, Oracle Health, Kansas City, Missouri).

Inclusion criteria contained (i) previous treatment restricted to surgery, (ii) subtotal resection on postoperative MRI not amenable for further resection, (iii) multidisciplinary-decision for adjuvant PRT and against institutional CyberKnife^®^-SRS or conventional RT, (iv) histologic confirmation of PA (FPA or NFPA).

Data collection was performed before each treatment and during follow-ups after 1, 6, 12 months, and every other year. Included data covered baseline characteristics before resection and PRT as well as last available follow-up reports. Each case contained (i) patient characteristics, (ii) tumor characteristics on imaging and histology, (iii) laboratory reports before and after PRT, (iv) radiotherapy plans, (v) follow-up imaging, (vi) ophthalmology and (vii) endocrinology reports before and after PRT, if available or conducted (Supplement 1). Data collection and assessment was performed from 08/2024 to 09/2025. Data storage and analysis were performed after pseudonymization. Ethics approval for retrospective collection and analysis was granted by UKHD Ethical Review Board. In accordance with the ethical approval missing data were followed up by contacting patients directly.

### Treatment characteristics and radiotherapy planning

Surgery was conducted either transnasal or via translamina terminalis approach. Initial re-resection due to regrowth was required in 54% of patients.

**Fig. 1:**
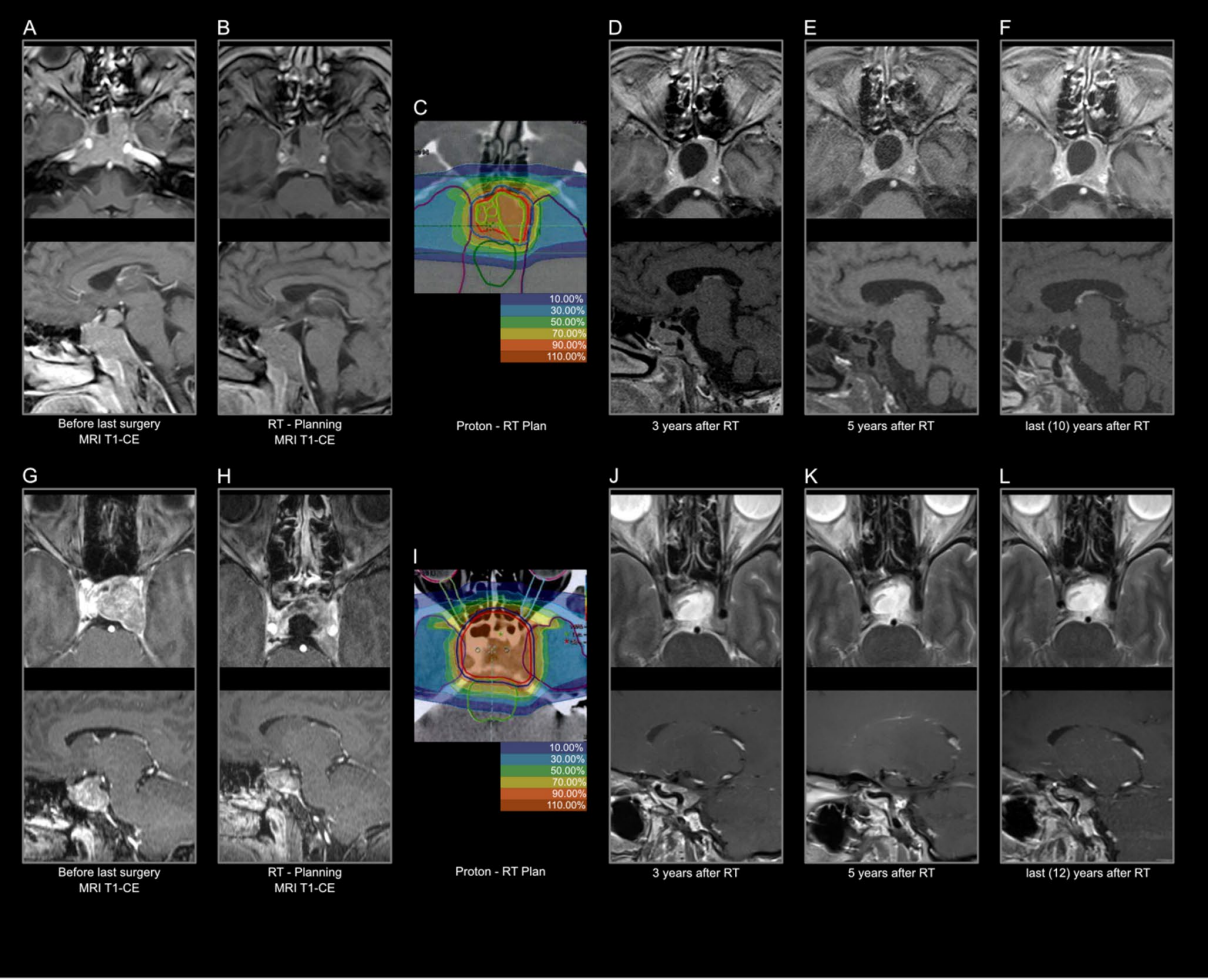
Tumor control of pituitary adenoma (PA) following adjuvant proton radiotherapy (PRT). Representative cases of two patients (PA-04: **A–F**; PA-05: **G–L**) with longest available MRI follow ups of 10 and 12 years, respectively, shown using MRI in axial and sagittal sections. (**A**,** G**): T1-weighted contrast-enhanced MRI before the last surgery. (**B**,** H**): MRI used for planning of additive PRT, depicting residual tumors as contrast-enhancing sellar lesions. (**C**,** I**): PRT treatment plan with isodose lines (% of total dose by color wash). Proton delivery via two opposing fields. Follow-up MRI (**D**,** J**): 3 years (**E**,** K**): 5 years (**F**,** L**): and last available post-PRT demonstrating durable tumor control and reduced tumor size in the sellar region

For PRT patients were immobilized in a thermoplastic head mask. A planning CT with 1 to 3 mm slice thickness was fused with contrast-enhanced T1- and T2-weighted MRI. The gross tumor volume (GTV) was defined as the postoperative residuum. For large, multicentric, or anatomically complex parasellar adenomas, a clinical target volume (CTV) was added to account for uncertainties in target delineation (Supplementary Table 1). The planning target volume (PTV) was generated by adding an isotropic 1 mm margin further adjusted for adjacent OARs. Proton median dose (D50) was prescribed to either the CTV (14/22) or if no to the GTV (8/22), delivered by active raster-scanning with two to three individually optimized, non-coplanar fields planned with a relative biological effectiveness (RBE) of 1.1. Daily image guidance was performed using two orthogonal x-ray representations of the skull to match position to the treatment plan. A robotic treatment table with a six-degree-of-freedom couch reduced positioning uncertainties to under 1 mm (Fig. 1). Dose coverage was achieved such that at least 95% of the target volume received between 96% and 107% of the prescribed dose. Dose constraints to OARs were applied according to QUANTEC recommendations [23]. PRT planning was conducted by either Syngo PT-Planning (Siemens, Erlangen, Germany) or RayStation (RaySearch Laboratories, Stockholm, Sweden). PRT plans were generated using robust optimization. Predefined setup and range uncertainty scenarios were considered.

### Study outcomes

Primary study outcome was the local tumor control rate (LCR) without regrowth on follow-up MRI for FPA and NFPA as well as hormone control rate (HCR) for FPA.

Secondary outcomes covered (i) radiation induced toxicity by CTCAE criteria, (ii) hormone control and required substitution/inhibition before and after PRT, (iii) vision improvement either as patient reported outcome or if available on visual acuity, /-field testing.

Hormone control for each pituitary axes was considered sufficient if basal endocrine testing was within the given reference. A change in visual acuity of 0.2 logarithm of Minimum Angle of Resolution (logMAR, two lines) was considered as improvement or worsening.

Visual field changes had to be declared as improving or worsening in ophthalmologic reports (Supplementary Table 1).

### Statistical analysis

LCR and HCR were estimated from the start of PRT to the last follow-up or time of relapse using the Kaplan–Meier estimator, with patients censored at their most recent assessment. For paired binary outcomes, normalization of hormone levels and requirement for endocrine medication before versus after PRT, one-sided McNemar’s tests were used. P-values < 0.05 were considered significant. Analyses were performed in R (version 4.5.2).

## Results

### Patient characteristics

Mean age at adjuvant PRT was 50.6 years. Male and female patients were equally represented. The median Karnofsky Performance Status before PRT was 90%.

All PAs demonstrated extrasellar extension preoperatively, frequently impinging on the optic apparatus (77%). Suprasellar and cavernous sinus involvement was universal, while sphenoidal extension was observed in 73%. The predominant PA subtype was NFPA, (72%); FPA were less common and included GH-secreting (18%), ACTH-secreting (5%), and prolactin-secreting (5%) adenoma.

Preoperative mass effects of PA outgrowth were common: visual disturbance in 17 (77%), cranial nerve involvement in 18 (83%), headache in 12 (55%); and hypopituitarism in 1 case (4%). Hormonally mediated symptoms were less frequent with acromegaly in 3 (14%), galactorrhea in 2 (9%), diminished libido in 1 (5%), and amenorrhea in 1 case (5%), consistent with the smaller subset of FPA.

After last resection and before adjuvant PRT, only minor relief of symptoms was reported. Residual visual disturbance persisted in 16 patients (75%) and 5 more were seen with hypopituitarism despite medication (overall 6/22, 27%). Headache symptoms persisted in 9 patients (41%). Cranial nerve deficits affected the optic nerve (73%), with less frequent involvement of the oculomotor (9%), trigeminal (5%), and olfactory (5%) nerves. 6 patients (28%) had no cranial nerve deficits. Hormonally mediated symptoms before PRT were uncommon (81%), with acromegaly and diminished libido each in 2 (9%) (Table 1).

**Table 1:** Baseline characteristics

Characteristics	Proton *n* = 22
Patient and tumor characteristics	
Age at radiotherapy (years) *Mean (SD.)*	50.6 (13.1)
Gender (female), *n (%)*	11 (50)
Karnofsky index before PRT (%) *Median (Min. – Max.)*	90 (60–100)
Tumor extension beyond sellar, *n (%)*	
Suprasellar	22 (100)
Cavernous	22 (100)
Sphenoid	16 (73)
Non-accessible	0 (0)
Adenoma subtype, *n (%)*	
NFPA	16 (73)
FPA	6 (27)
ACTH	1 (5)
GH	4 (18)
Prolactin	1 (5)
**Symptoms before surgery**	
Mass-effect symptoms, *n (%)*	
None	0 (0)
Visual disturbance	17 (77)
Cranial nerve affection	18 (82)
Hypopituitarism	1 (5)
Headache	12 (55)
Hormone-related symptoms, *n (%)*	
None	17 (77)
Galactorrhea	2 (9)
Acromegaly	3 (14)
Diminished libido	1 (5)
Amenorrhea	1 (5)
**Symptoms before radiotherapy**	
Residual symptoms post-resection, *n (%)*	
None	4 (18)
Visual disturbance	16 (73)
Hypopituitarism	6 (27)
Headache	9 (41)
Affected cranial nerves, *n (%)*	
None	6 (28)
Opticus	16 (73)
﻿Olfactory	1 (5)
Oculomotors	2 (9)
Trigeminus	1 (5)
Hormone-related symptoms, *n (%)*	
None	18 (81)
Galactorrhea	0 (0)
Acromegaly	2 (9)
Diminished libido	2 (9)
Amenorrhea	0 (0)

Abbreviations: Min, Minimum; Max, Maximum; SD, Standard deviation; ACTH, Adrenocorticotropic hormone; GH, Growth hormone

Data are presented as number (%) or mean (standard deviation), median (minimum – maximum)

### Treatment characteristics

Patients had a median of 2 resections prior to RT. The indication for adjuvant RT was residual tumor in all cases, with 14 patients (64%) treated for radiographic residual disease alone and 8 patients (36%) for a combination of residual tumor and persisting hormone excess. PRT was selected for residual macroadenomas (maximum adenoma diameter > 10 mm) with irregular extrasellar extension and carotid artery contact in all cases, and additional contact to the optic apparatus or brain parenchyma in 68% of cases. Planning imaging for each case is shown in the supplementary Table 2. Postoperative residual tumor volume had a median of 15.0 cm³ (range, 0.7–53.4). The median interval between last surgery and start of PRT was 4 months. In 8 patients PRT-dose was prescribed to a median GTV of 9.2 cm³, while a CTV was required in 14 patients, with a median volume of 34.4 cm³.

The median prescribed dose (Dmedian, D50.0) was 52,2 Gy (RBE) for NFPA (range, 50.4–54.0) and 50.4 in FPA (range, 50.4–54.0) delivered in a median of 28 fractions.

Dosimetry to the optic apparatus remained below predefined dose constraints. The median maximum doses (Dmax, D0.03) to the right-, left optic nerve and optic chiasm were 50.2 Gy (RBE) (Table 2, Supplementary Table 1).

**Table 2:** Treatment characteristics

Characteristics	Proton *n* = 22
Surgery	
Number of resections *Median (Min. – Max).*	2 (1–4)
Indication for adjuvant RT, *n (%)*	
Residual tumor only	14 (64)
Persisting hormone levels only	0 (0)
Both	8 (36)
Postoperative criteria for adjuvant PRT	
Macroadenoma*	22 (100)
Irregular, complex residual PA shape	22 (100)
Contact with optic system	15 (68)
Contact with carotid artery	22 (100)
Contact with brain parenchyma	15 (68)
Postoperative residual adenoma volume [cm^3^] *Median (Min.-Max.)*	15.0 (0.7–53.4)
**Additive proton radiotherapy**	
Median time to radiotherapy (From last surgery; in months) *Median (Min. – Max*.)	4 (1–12)
Volume of prescribed target [cm^3^]** *Median (Min.-Max.)*	
GTV	9.2 (2.2–53.4)
CTV	34.4 (3.3–75.2)
Prescribed median dose [Gy RBE] *Median (Min. – Max.)*	
NFPA	52.2 (50.4–54.0)
FPA	50.4 (50.4–54.0)
Applied fractions, *n* *Median (Min. – Max.)*	28 (25–30)
**Dosimetry at the optic system**	
Applied Dmax right optic nerve [Gy RBE] *Median (Min. – Max.)*	50.4 (41.5–52.4)
Applied Dmax left optic nerve [Gy RBE] *Median (Min. – Max.)*	50.2 (21.6–52.8)
Applied Dmax optic chiasm [Gy RBE] *Median (Min. – Max.)*	50.2 (46.1–52.6)

Abbreviations: Min, Minimum; Max, Maximum; PRT, Proton radiotherapy; GTV, Gross tumor volume; PTV, Planning target volume; Dmean, Mean dose; Dmax, Maximum dose; RBE, Relative biological effectiveness

*Macroadenoma as a residual adenoma with a maximum diameter > 10 mm

**Dose prescription to GTV (8/22) or CTV (14/22)

Data are presented as number (%) or median (minimum – maximum). Dmedian (median dose) as D50.0 and Dmax as D0.03

### Radiation-related toxicity

Radiation-related toxicity was mild and predominantly early in onset. Fatigue was most frequent, reported as grade 1 in 55% and grade 2 in 14%, mainly occurring during PRT or within the first 3 months thereafter. Headache occurred in 41% as grade 1 and 14% as grade 2, almost exclusively during treatment or in the acute phase. Transient grade 1 skin reactions and focal hair loss were documented in 23% and 18% of patients, respectively during or in the acute post-treatment period. Reported visual disturbances were uncommon and low grade, with grade 1 changes in 14% and grade 2 changes in 5%, occurring early and transient. Less frequent events included grade 1 hearing disturbance (*n* = 1, 5%), grade 1 mucositis (*n* = 1, 5%), and grade 1 vertigo in 3 patients (14%). No secondary malignancy was observed. Complete toxicity reporting was available in all patients up to 3 months and in 21 patients (95%) beyond 6 months (Table 3).

**Table 3:** Toxicity of adjuvant proton radiotherapy

Toxicity, CTCAE (*n* °)	Proton *n* = 22				
Time of onset (Months)	Total	Early-Onset		Late-Onset	
		During PRT	Acute < 3	Subacute ≥ 3 - <6	Late ≥ 6
Available follow-up reports per time point, *n (%)*		22 (100)	22 (100)	22 (100)	21 (95)
Vision changes, *n (%)*					
1	3 (14)	1 (5)	2 (9)	0 (0)	0 (0)
2	1 (5)	0 (0)	1 (5)	0 (0)	0 (0)
Skin changes, *n (%)*					
1	5 (23)	5 (23)	0 (0)	0 (0)	0 (0)
Focal hair Loss, *n (%)*					
1	4 (18)	2 (9)	2 (9)	0 (0)	0 (0)
Fatigue, *n (%)*					
1	12 (55)	9 (41)	3 (14)	0 (0)	0 (0)
2	3 (14)	2 (9)	1 (5)	0 (0)	0 (0)
Headache, *n (%)*					
1	9 (41)	9 (41)	0 (0)	0 (0)	0 (0)
2	3 (14)	2 (9)	1 (5)	0 (0)	0 (0)
Hearing disturbance, *n (%)*					
1	1 (5)	0 (0)	1 (5)	0 (0)	0 (0)
Mucositis, *n (%)*					
1	1 (5)	1 (5)	0 (0)	0 (0)	0 (0)
Vertigo, *n (%)*					
1	3 (14)	2 (9)	1 (5)	0 (0)	0 (0)

Abbreviations: PRT, Proton radiotherapy; CTCAE, Common terminology criteria for adverse events

Data are presented as number (%) for each CTCAE grade (°) in each category of reported toxicity

Toxicities categorized by CTCAE v5.0: Hair Loss: Skin and subcutaneous tissue disorders (focal alopecia), Skin changes: Skin and subcutaneous tissue disorders (Photosensitivity), Vision changes: Eye disorders (Unspecified, Optic nerve disorder, Vision decreased), Fatigue: General disorders and administration site conditions (Fatigue), Headache: Nervous system disorders (Headache), Hearing disturbance: Ear and labyrinth disorders (Hearing impaired), Mucositis: Gastrointestinal disorders (Mucositis oral), Vertigo: Ear and labyrinth disorders (Vertigo), Cognitive deficiency: Nervous system disorders (Cognitive disturbance, concentration impairment)

### Study outcomes

After a median follow-up of 65 months, adjuvant PRT achieved excellent PA-control in FPA and NFPA. The 3- and 5-year LCR were both 100%. The HCR at 3- and 5-years in FPA was as well 100% although only 6 cases were at risk (Table 4).

Radiographic tumor regression was observed in all cases. Endocrine outcomes reached favorable results. For the prolactin axis, high prolactin levels were observed in four patients after surgery related to the stalk effect. The proportion of patients with a stable prolactin axis increased from 82% after surgery to 100% after PRT. In the somatotropin axis, hormone inhibition was not required despite elevated somatotropin levels after surgery, which all reached stable function after PRT (from 82% to 100%). The most pronounced endocrine effect was seen in the glucocorticoid axis. The number of patients requiring hormone replacement decreased from 18 (82%) after surgery to 11 (50%) after PRT (*p* = 0.02). A stable glucocorticoid status increased from 73% to 100% (*p* = 0.04). Thyroid hormone replacement increased from 36% to 50% (*p* = 0.22) with stable thyrotropin axis function in 21 (96%) versus 22 (100%) patients (*p* = 0.50). Gonadotropin replacement was required in 14% of patients after PRT compared to none after surgery (*p* = 0.12), while stable gonadotropin status increased from 73% to 96% (*p* = 0.07). Vasopressin replacement remained unchanged before and after PRT, with stable vasopressin status increasing from 91% to 100% (*p* = 0.24). Newly diagnosed secondary hypopituitarism after PRT occurred in 1 patient. Taken together, the addition of PRT did neither result in unstable hormone axes, nor increase the incidence of hypopituitarism (Table 4, Supplementary Table 1).

Although present transient vision changes, overall visual outcomes were favorable in long-term follow ups. Based on patient-reported vision, 6 patients (27%) had unimpaired vision both before and after PRT, 9% reported stable pre-existing impairment, and 64% reported an improvement. No patient reported a worsening experienced after PRT. Objective best-corrected visual acuity was stable in 12 patients (55%), improved in 3 (14%), and worsened in 2 (9%). Of the two patients with worsened visual acuity, one case was attributed to radiation-induced optic neuropathy, whereas the other was not radiation related and due to chronic glaucoma diagnosed prior to PA treatment. Visual field testing showed stability in 41% and improvement in 36%, with no recorded deterioration; Ophthalmologic follow-up data were unavailable for 5 patients (23%); 3 of these had no visual impairment prior to treatment (Table 4, Supplementary Table 1).

**Table 4:** Outcomes after adjuvant proton radiotherapy

Outcomes	Proton *n* = 22		
Follow up time (Months) *Median (Min.-Max.)*	65 (3-146)		
**Local tumor control rate in NFPA and FPA**			
* Available n at risk*,* %*	22 (100%)		
3-Year LCR, *% (95-CI)*	100 (100–100)		
5-Year LCR, *% (95-CI)*	100 (100–100)		
**Hormone control rate in FPA**			
* Available n at risk*,* %*	6 (100%)		
3-Year HCR, *% (95-CI)*	100 (100–100)		
5-Year HCR, *% (95-CI)*	100 (100–100)		
**Hormone status per axis**	**After surgery**	**After PRT**	*p-value*
Prolactin			
Hormone Inhibition, *n (%)*	0 (0)	0 (0)	-/-
Stable, *n (%)*	18 (82)	22 (100)	0.07
Somatotropin axis			
Hormone inhibition, *n (%)*	0 (0)	0 (0)	-/-
Stable, *n (%)*	18 (82)	22 (100)	0.07
Glucocorticoid axis			
Hormone replacement, *n (%)*	18 (82)	11 (50)	**0.02**
Stable, *n (%)*	17 (77)	22 (100)	**0.04**
Thyrotropin axis			
Hormone replacement, *n (%)*	8 (36)	11 (50)	0.22
Stable, *n (%)*	21 (96)	22 (100)	0.50
Gonadotropin axis			
Hormone replacement, *n (%)*	0 (0)	3 (14)	0.12
Stable, *n (%)*	16 (73)	21 (96)	0.07
Vasopressin axis			
Hormone replacement, *n (%)*	2 (9)	2 (9)	-/-
Stable, *n (%)*	20 (91)	22 (100)	0.24
Not available, *n (%)*	0 (0)	0 (0)	-/-
**Vision changes**			
Patient-reported vision outcome			
Not impaired before and after PRT	6 (27)		
Stable	2 (9)		
Worsened	0 (0)		
Improved	14 (64)		
Not available	0 (0)		
Visual acuity*			
Stable	12 (55)		
Worsened	2 (9)		
Improved	3 (14)		
Not available	5 (23)		
Vision field			
Stable	9 (41)		
Worsened	0 (0)		
Improved	8 (36)		
Not available	5 (23)		
Secondary blindness	0 (0)		

Abbreviations: Min, Minimum; Max, Maximum; LCR, Local tumor control rate; HCR, Hormone control rate; 95-CI, 95% Confidence interval; PRT, Proton radiotherapy

Data are presented as number (%), percentage per time point (95%-Confidence interval) or median (minimum – maximum). Statistical comparison of hormone control before and after PRT conducted by one-sided McNemar tests. P-values < 0.05 regarded as significant

Comprehensive list of full patient details in supplementary Fig. 1

*Improvement in visual acuity reported if best-corrected visual acuity reaches ≥ 0.2 logarithm of Minimum angle of resolution (logMAR, two lines). 2 Patients identified with worsened visual acuity, one with possible radiation induced optic neuropathy and one with chronic glaucoma diagnosed before PA treatment

## Discussion

In this retrospective cohort study, adjuvant PRT demonstrated an effective and safe treatment strategy for complex PA with large residual tumor volumes in vulnerable locations.

### Role of adjuvant PRT

Subtotal resection in pituitary macroadenoma frequently necessitates efficacious adjuvant treatment. Postoperative photon-based RT already demonstrated long-term local control rates using modern intensity modulated radiotherapy (IMRT) or SRS [6, 24–26]. However, few complex residual macroadenoma may remain challenging for these techniques due to large volumes, irregular shapes, extrasellar extension, cavernous sinus invasion and direct contact to the optic system, carotid artery or brain parenchyma. Single-fraction or fractionated SRS may be limited by tumor size, irregular extension, or insufficient distance to the optic apparatus. Kim et al. [27] and Sathe et al. [25] reported favorable local control for small residual PA adjacent to, but not contacting, the optic pathways. Shen et al. [28] observed optic neuropathy in 12% of cases and identified optic apparatus contact and tumor volume > 3.5 cm³ as risk factors. Recent IMRT/VMAT series mainly included relatively small PA and used conventional fractionation to reduce the risk of OAR toxicity and optic neuropathy attributed to high dose of single fraction in SRS. Hemaidia et al. [29] reported difficulty maintaining optic pathway doses below 54 Gy in challenging locations, Brand et al. [30] required transition from VMAT to PRT to meet dose constraints in selected cases, and Scheick et al. [24] treated rather small remnants close to OARs, with a median volume of 1.9 cm³. Thus, large, irregular, or perioptic residual adenomas may exceed the practical limits of SRS or conventional RT, supporting consideration of PRT when OAR sparing or integral dose reduction is critical (Table 5). The present PRT study maintained perfect local tumor control within set dose constraints and despite direct contact to OARs. This suggests that presented criteria applied in the unicentric decision-making process may guide the selection of PRT over photon-based techniques to maintain PA control in challenging cases.

**Table 5:** Recent radiotherapy studies for residual or recurrent pituitary adenoma

Study	Year	Patient number	RT technique	Median tumor volume [cm^3^]	Prescribed does [Gy]	LCR (Median FU time)	Secondary hypopituitarism	RT-related vision impairment
Present manuscript	/	22	PRT	15.0	50.4–54.0	100% (5.4 years)	10%	5% (1/22)
Kim et al.	2025	83	SRS	1.7	8.0–25.0	100% (4.7 years)	14%	0% (0/83)
Sathe et al.	2023	75	FSRT	7.6	50.4	91.2 (5.0 years)	15%	4% (3/72)
Shen et al.	2018	41	SRS	5.5	8.0	92.6% (10.0 years)	17%	12% (5/51)
Hemaidia et al.	2026	35	RT (VMAT)	3.9	54.0	96.0% (5.0 years)	15%	3% (1/33)
Scheick et al.	2016	116	RT (IMRT)	1.9	45.0–54.0	96.0% (10.0 years)	26%	0 (0/116)
Brand et al.	2025	122	RT/PRT	Na	50.0-59.4	95.0% (5.0 years)	40% *	< 3 (Na)

Abbreviations: RT, radiotherapy; VMAT, volumetric modulated arc therapy; IMRT, intensity-modulated radiotherapy; PRT, proton radiotherapy; LCR, Local tumor control rate

*Hypopituitarism not differentiated as radiation-related or surgery-related

### Toxicity of PRT

Observed radiation-related side effects in earlier series limited the application of radiation in the vulnerable sellar region [31, 32]. Modern precision radiotherapy substantially reduced side effects to an overall minor toxicity. This was again the case in the present PRT cohort study showing a low toxicity burden with most frequently headache and fatigue and without any CTCAE grade 4 or higher events.

Reported rates of radiation-induced secondary hypopituitarism declined to under 25% in recent studies mostly affecting the glucocorticoid and thyroid axis [14, 27]. The risk of RT-related hypopituitarism was either associated with the extend of adenoma tissue irradiation or treatment sequence with postoperative RT increasing secondary hypopituitarism by a minor fraction of 5–10% additionally to 30–40% after surgery [33, 34]. Reducing target-volumes from whole sellar to adenoma coverage contributed to the reduction of hypopituitarism while preserving low progression rates. Furthermore, sufficient hormone replacement contributed to a high rate of overall hormone control regardless of surgery or RT.

These results differ from cohorts receiving cranial PRT. In a large pediatric and young adult cohort, Vatner et al. [35] demonstrated that endocrine deficiencies increase with higher median dose to the composite hypothalamus–pituitary axis, younger age, and longer follow-up. At a median follow-up of 4.4 years, the 4-year incidence of any hormone deficiency was ~ 49%, with growth hormone deficiency being most frequent (~ 37%). Thus, longer observation periods are associated with higher detection rates of endocrine insufficiency, underscoring the need for long-term endocrine follow-up. Brand et al. [30] outlined a secondary hypopituitarism rate of about 40% after RT for PA. This high rate might be due to the included clinical heterogeneity of each cohort covering salvage, post operative and definitive situations, different RT-modalities and may not distinguish between postoperative hypopituitarism and additional hypopituitarism after radiotherapy.

Advances in RT technique have as well reduced the risk for radiation-induced visual disturbance in PA. Early series using two-dimensional, large-field techniques caused concern to induce damage to the optic chiasm and nerves attributed to increased single fraction doses of up to 2.5 Gy and a Dmax of exceeding 54 Gy at the optic nerves or chiasm [36, 37]. Contemporary series of pituitary irradiation with image-guided techniques report visual deficits in less than 3% of patients, even when remnants are adjacent to the optic system [26, 38]. Several studies reported not only preservation but also improvement of vision impairment [10, 11]. We underscore patient-reported vision improvement in 64% of all cases which was verified on ophthalmologic testing by improved visual acuity or improved vision fields in 44% of patients. These findings highlight the correlation of improved clinical outcomes to the superior PRT dose conformity in complex macroadenoma. Nevertheless, long-term follow-up remains essential to detect delayed radiation-associated side effects.

### Limitations

Present retrospective design is inherently subject to selection bias and unmeasured confounding. The sample size is small, which restricts the precision of estimates for rare events. Endocrine and ophthalmologic outcomes were not standardized and comprehensive functional testing, including dynamic endocrine testing where indicated was absent, limiting the interpretation of functional outcome measures.

## Conclusion

Adjuvant PRT is a highly effective treatment strategy for residual, complex PA, providing excellent tumor control with a low toxicity burden, favorable hormonal control, and preservation or improvement of visual function.

## Supplementary Information

Below is the link to the electronic supplementary material.

Supplementary Material 1

Supplementary Material 2

## Data Availability

Presented clinical datasets will be made available up on reasonable request from the corresponding author.
