# Supplementary material for: Long-term outcomes of adjuvant proton radiotherapy (PRT) for residual pituitary adenoma (PA) in adults – a retrospective, single institute experience
Source: J Neurooncol. 2026 Jun 12;178(2):60. doi: 10.1007/s11060-026-05669-2 (PMC13260228; doi:10.1007/s11060-026-05669-2)
Supplement: Supplementary file 2 — Supplementary Material 2 [file 11060_2026_5669_MOESM2_ESM.docx]

**Supplement**

**Supplementary table 1:** Individual patient characteristics included in presented study

| **Patient-ID** | **PA-Subtype** | **No. of resections** | **Residual GTV**  **[cm^3^]** | **Pre-PRT**  **hormone levels** | | | | | | **Prescribed Dmedian**  **[Gy RBE]** | **Optic system**  **Dmax [Gy RBE]** | | | **Follow up**  **Time**  **[months]** | **Post-PRT hormone levels** | | | | | | **Vision changes** | | |
| --- | --- | --- | --- | --- | --- | --- | --- | --- | --- | --- | --- | --- | --- | --- | --- | --- | --- | --- | --- | --- | --- | --- | --- |
|  |  |  |  | *P* | *SA* | *GA* | *TA* | *GoA* | *VaA* |  | *ON (left)* | *ON (right)* | *OC* |  | *P* | *SA* | *GA* | *TA* | *GoA* | *VA* | *VA*  *(right eye)* | *VA*  *(left eye)* | *VF* |
| **PA-01** | GH | 4 | 2.2 | 0 | **+** | 0 (S) | 0 | 0 | 0 | 54,0 (GTV) | 50.5 | 50.8 | 52.2 | 59 | 0 | 0 | 0 (S) | 0 | 0 | 0 | 0 | 0 | 0 |
| **PA-02** | Ns | 1 | 11.6 | 0 | 0 | 0 (S) | 0 | 0 | 0 | 52,2 (GTV) | 42.4 | 49.9 | 50.0 | 52 | 0 | 0 | 0 | 0 | 0 | 0 | 0 (Ni) | 0 (Ni) | 0 (Ni) |
| **PA-03** | Ns | 1 | 24.5 | 0 | 0 | 0 (S) | 0 (S) | **-** | **-** (S) | 52,2 (CTV) | 50.0 | 50.4 | 50.4 | 72 | 0 | 0 | 0 (S) | 0 (S) | 0 | 0 (S) | 0 | 0 | 0 |
| **PA-04** | Ns | 3 | 34.8 | 0 | 0 | 0 (S) | 0 (S) | 0 | 0 | 50,4 (CTV) | 49.7 | 49.8 | 49.0 | 137 | 0 | 0 | 0 | 0 (S) | **-** | 0 | 0 | 0 | 0 |
| **PA-05** | Ns | 3 | 8.1 | 0 | 0 | **-** (S) | 0 (S) | **-** | 0 | 52,0 (CTV) | 52.8 | 52.0 | 52.6 | 146 | 0 | 0 | 0 (S) | 0 (S) | 0 (S) | 0 | **+** (Δ 0.2) | 0 | **+** |
| **PA-06** | Ns | 1 | 23.6 | 0 | 0 | 0 (S) | 0 | 0 | 0 | 54,0 (CTV) | 51.0 | 51.0 | 51.0 | 10 | 0 | 0 | 0 | 0 | 0 | 0 | 0 (Ni) | **-** (Δ 0.2)^1^ | 0 (Ni) |
| **PA-07** | Ns | 2 | 22.3 | **+** | 0 | 0 (S) | 0 (S) | **-** | 0 | 54,0 (CTV) | 51.9 | 51.8 | 52.0 | 131 | 0 | 0 | 0 | 0 (S) | 0 | 0 | 0 | 0 | **+** |
| **PA-08** | Ns | 2 | 22.0 | **+** | 0 | **-** (S) | **-** | **-** | **-** (S) | 50,4 (CTV) | 51.2 | 51.0 | 51.4 | 144 | 0 | 0 | 0 (S) | 0 (S) | 0 | 0 (S) | **+** (Δ 0.2) | **+** (Δ 0.4) | **+** |
| **PA-09** | ACTH | 2 | 5.3 | 0 | 0 | 0 | 0 | **-** | 0 | 52,0 (CTV) | 50.6 | 50.7 | 49.0 | 114 | 0 | 0 | 0 (S) | 0 (S) | 0 (S) | 0 | 0 | **-** (Δ 0.6)^2^ | 0 |
| **PA-10** | Ns | 2 | 53.4 | 0 | 0 | 0 (S) | 0 | 0 | 0 | 50,4 (GTV) | 50.4 | 50.4 | 50.5 | 135 | 0 | 0 | 0 (S) | 0 (S) | 0 | 0 | 0 | 0 | **+** |
| **PA-11** | GH | 1 | 47.3 | 0 | 0 | 0 (S) | 0 (S) | 0 | 0 | 54,0 (CTV) | 52.2 | 51.7 | 51.8 | 18 | 0 | 0 | 0 (S) | 0 (S) | 0 | 0 | 0 | 0 | 0 |
| **PA-12** | Ns | 3 | 7.3 | 0 | 0 | 0 | 0 | 0 | 0 | 52,2 (CTV) | 49.3 | 49.3 | 48.9 | 106 | 0 | 0 | 0 | 0 (S) | 0 | 0 | 0 | 0 | 0 |
| **PA-13** | Ns | 2 | 6.2 | 0 | 0 | **+** (S) | 0 (S) | **-** | 0 | 50,4 (GTV) | 49.3 | 49.2 | 48.2 | 69 | 0 | 0 | 0 (S) | 0 | 0 | 0 | Na | Na | Na |
| **PA-14** | Ns | 1 | 6.8 | **+** | 0 | 0 (S) | 0 (S) | 0 | 0 | 50,4 (GTV) | 49.4 | 41.5 | 49.1 | 84 | 0 | 0 | 0 (S) | 0 | 0 | 0 | Na (Ni) | Na (Ni) | Na (Ni) |
| **PA-15** | P | 1 | 2.9 | 0 | 0 | 0 (S) | 0 | 0 | 0 | 50,4 (GTV) | 21.6 | 49.3 | 46.1 | 83 | 0 | 0 | 0 | 0 | 0 | 0 | Na (Ni) | Na (Ni) | Na (Ni) |
| **PA-16** | Ns | 1 | 3.6 | 0 | 0 | 0 (S) | 0 (S) | 0 | 0 | 52,2 (CTV) | 50.5 | 51.6 | 51.9 | 61 | 0 | 0 | 0 (S) | 0 (S) | 0 (S) | 0 | 0 (Ni) | 0 (Ni) | 0 (Ni) |
| **PA-17** | Ns | 1 | 15.2 | **+** | 0 | 0 (S) | 0 | 0 | 0 | 54,0 (GTV) | 52.0 | 52.4 | 52.3 | 3 | 0 | 0 | 0 | 0 | 0 | 0 | Na | Na | Na |
| **PA-18** | GH | 1 | 16.6 | 0 | **+** | **+** (S) | 0 | 0 | 0 | 50,4 (CTV) | 50.0 | 49.6 | 49.9 | 44 | 0 | 0 | 0 (S) | 0 | 0 | 0 | 0 | 0 | **+** |
| **PA-19** | Ns | 2 | 3.0 | 0 | **+** | **+** (S) | 0 | 0 | 0 | 50,4 (CTV) | 50.5 | 49.0 | 49.5 | 16 | 0 | 0 | 0 | 0 (S) | 0 | 0 | Na (Ni) | Na (Ni) | Na (Ni) |
| **PA-20** | Ns | 1 | 0.7 | 0 | 0 | 0 | 0 | 0 | 0 | 50,4 (CTV) | 48.8 | 49.4 | 49.8 | 32 | 0 | 0 | 0 | 0 | 0 | 0 | 0 | **+** (Δ 0.4) | **+** |
| **PA-21** | GH | 2 | 14.8 | 0 | **+** | 0 (S) | 0 | 0 | 0 | 50,4 (GTV) | 43.6 | 50.0 | 49.9 | 42 | 0 | 0 | 0 | 0 | 0 | 0 | 0 | 0 | **+** |
| **PA-22** | Ns | 3 | 29.3 | 0 | 0 | 0 | 0 | 0 | 0 | 54,0 (CTV) | 49.6 | 51.7 | 51.6 | 18 | 0 | 0 | 0 | 0 | 0 | 0 | 0 | 0 | **+** |

*Abbreviations: ACTH, Adrenocorticotropic hormone; CTV, Clinical target volume; Dmax, Maximum dose; Dmean, Mean dose; GA, Glucocorticoid axis; GH, Growth hormone; GTV, Gross tumor volume; GoA, Gonadotropin axis; ID, Identifier; Na, Not available; Ni, Not impaired before PRT; No., Number; Ns, Non-secreting; OC, Optic chiasm; ON, Optic nerve; P, Prolactin; PA, Pituitary adenoma; PRT, Proton radiotherapy; RBE, Relative biological effectiveness; S, Substituted; SA, Somatotropin axis; TA, Thyrotropin axis; VA, Visual acuity; VaA, Vasopressin axis; VF, Visual field*

Categorical classification of reported hormone levels (0: normal, +: increased, -: decreased), visual acuity and visual fields (0: stable, +: improved, -: worsened). Improvement in Visual acuity reported if best-corrected visual acuity reaches ≥ 0.2 logarithm of Minimum angle of resolution shown as delta (Δ) pre-/post-PRT (logMAR, two lines).

Comments: 1. Possible RION without clinical symptoms or contrast-enhanced left ON on MRI (no relevant comorbidities with increased risk for vision deterioration); 2. Decrease in visual acuity due to chronic glaucoma with macular degeneration diagnosed before PA treatment (Glaucoma diagnosis: 2004, Radiotherapy 2013),

**Supplementary table 2:** Description of the criteria applied for selecting proton radiotherapy as the radiation modality in each included case

| **Patient ID** | **PRT-planning images after surgery: MRI or CT** | **Indication criteria for PRT** |
| --- | --- | --- |
| PA-01 |  | **Hardy**: IVE  **Knosp**: 2  **Criteria**:   - Size: Macroadenoma *(Max diameter > 10mm*) - Shape: Irregular (*Complex boarder configuration alongside sellar wall into cavernous sinus. Delineable borders allowed target dose prescription to the GTV*) - Expansion: Extrasellar *(suprasellar, cavernous, sphenoid)* - Contact to OARs: Optic nerve, carotid artery (*In direct contact with right carotid artery and right optic nerve*) |
| PA-02 |  | **Hardy**: IVE  **Knosp**: 4  **Criteria**:   - Size: Macroadenoma (*Max diameter > 10mm*) - Shape: Irregular (*Complex boarder configuration due to diffuse extrasellar extension reaching right temporal lobe parenchyma. Delineable borders allowed target dose prescription to the GTV.)* - Expansion: Extrasellar (*suprasellar, cavernous*) - Contact to OAR: Optic nerve, carotid artery, brain parenchyma (*Encasement of right* *carotid artery and right optic nerve, direct contact to temporal lobe parenchyma*) |
| PA-03 |  | **Hardy**: IVE  **Knosp**: 3A  **Criteria**:   - Size: Macroadenoma (*Max diameter > 10mm*) - Shape: Irregular (*Complex boarder configuration due to diffuse extrasellar extension not clearly to delineate on CT, no MRI due to claustrophobia))* - Expansion: Extrasellar (*suprasellar, cavernous, sphenoid*) - Contact to OAR: Optic nerve, carotid artery *(In direct contact with right carotid artery and right optic nerve*) |
| PA-04 |  | **Hardy**: IVE  **Knosp**: 4  **Criteria**:   - Size: Macroadenoma (*Max diameter > 10mm*) - Shape: Irregular (*Complex boarder configuration due to diffuse extrasellar extension reaching temporal lobe parenchyma and ventral brain stem)* - Expansion: Extrasellar (*suprasellar, cavernous*) - Contact to OAR: Optic nerve, carotid artery, brain parenchyma (*Encasement of left* *carotid artery and left optic nerve, direct contact to temporal lobe and ventral brainstem parenchyma*) |
| PA-05 |  | **Hardy**: IVE  **Knosp**: 3A  **Criteria**:   - Size: Macroadenoma (*Max diameter > 10mm*) - Shape: Irregular (*Complex boarder configuration alongside sellar wall into cavernous sinus*) - Expansion: Extrasellar (*suprasellar, cavernous, sphenoid*) - Contact to OARs: Carotid artery (*In direct contact with right carotid artery*) |
| PA-06 |  | **Hardy**: IVE  **Knosp**: 4  **Criteria**:   - Size: Macroadenoma (*Max diameter > 10mm*) - Shape: Irregular (*Complex border configuration due to diffuse extrasellar extension, invading both sides of the cavernous sinus and reaching both temporal lobes*) - Expansion: Extrasellar (*suprasellar, cavernous, sphenoid)* - Contact to OAR: Optic nerve, carotid artery, (*Encasement of both carotid arteries, adjacent to both optic nerves and temporal lobes*) |
| PA-07 |  | **Hardy**: IVE  **Knosp**: 3A  **Criteria**:   - Size: Macroadenoma (*Max diameter > 10mm*) - Shape: Irregular (*Complex border configuration due to right extrasellar residuum within the cavernous sinus and alongside the right temporal lobes*) - Expansion: Extrasellar (*suprasellar, cavernous, sphenoid)* - Contact to OAR: Optic nerve, carotid artery, brain parenchyma (*In direct contact with right carotid artery, optic nerve and temporal lobe*) |

***Continued:* Supplementary table S2**

| Patient ID | PRT-planning images after surgery: Contrast enhanced MRI or CT | Indication criteria for PRT |
| --- | --- | --- |
| PA-08 |  | **Hardy**: IVE  **Knosp**: 4  **Criteria**:   - Size: Macroadenoma (*Max diameter > 10mm*) - Shape: Irregular (*Complex border configuration due to extrasellar expansion in the cavernous sinus reaching the left temporal lobe*) - Expansion: Extrasellar (*suprasellar, cavernous, sphenoid)* - Contact to OAR: Optic nerve, carotid artery, brain parenchyma (*Encasement of both carotid arteries, in direct contact to left optic nerve and left temporal lobe*) |
| PA-09 |  | **Hardy**: IVE  **Knosp**: 4  **Criteria**:   - Size: Macroadenoma (*Max diameter > 10mm*) - Shape: Irregular (*Complex border configuration due to extrasellar expansion in particular in the right cavernous sinus*) - Expansion: Extrasellar (*suprasellar, cavernous, sphenoid)* - Contact to OAR: Optic nerve, carotid artery, brain parenchyma (*Encasement of both carotid arteries, in direct contact to left optic nerve and left temporal lobe*) |
| PA-10 |  | **Hardy**: IVE  **Knosp**: 4  **Criteria**:   - Size: Macroadenoma (*Max diameter > 10mm*) - Shape: Irregular (*Complex border configuration due to diffuse extrasellar extension reaching both temporal lobes and ventral brainstem. Proximity to OARs in all directions warranted omission of a separate CTV and thus equated CTV with GTV)* - Expansion: Extrasellar (*suprasellar, cavernous, sphenoid*) - Contact to OAR: Optic nerve, carotid artery, brain parenchyma (*Encasement of both carotid arteries and left optic nerve, direct contact to right optic nerve, both temporal lobes and ventral brainstem*) |
| PA-11 |  | **Hardy**: IVE  **Knosp**: 4  **Criteria**:   - Size: Macroadenoma (*Max diameter > 10mm*) - Shape: Irregular (*Complex border configuration due to diffuse extrasellar extension reaching left temporal lobe parenchyma and ventral brainstem)* - Expansion: Extrasellar (*suprasellar, cavernous, sphenoid*) - Contact to OAR: Optic nerve, carotid artery, brain parenchyma (*Encasement of left carotid artery and left optic nerve, direct contact to right carotid artery, right optic nerve, both temporal lobes and ventral brainstem*) |
| PA-12 |  | **Hardy**: IVE  **Knosp**: 1  **Criteria**:   - Size: Macroadenoma (*Max diameter > 10mm*) - Shape: Irregular (*Complex border configuration due to diffuse extrasellar extension alongside the right carotid artery)* - Expansion: Extrasellar (*suprasellar, cavernous*) - Contact to OAR: Carotid artery, optic nerve (*Direct contact to right carotid artery, right optic nerve*) |
| PA-13 |  | **Hardy**: IVE  **Knosp**: 2  **Criteria**:   - Size: Macroadenoma (*Max diameter > 10mm*) - Shape: Irregular (*Complex border configuration due to diffuse extrasellar extension alongside the right carotid artery. Delineable borders allowed target dose prescription to the GTV)* - Expansion: Extrasellar (*suprasellar, cavernous*) - Contact to OAR: Carotid artery (*Direct contact to right carotid artery*) |
| PA-14 |  | **Hardy**: IVE  **Knosp**: 3A  **Criteria**:   - Size: Macroadenoma (*Max diameter > 10mm*) - Shape: Irregular (*Complex border configuration due to diffuse extrasellar extension reaching the left inferior frontal lobe parenchyma. Delineable borders allowed target dose prescription to the GTV)* - Expansion: Extrasellar (suprasellar, cavernous) - Contact to OAR: Optic nerve, carotid artery, brain parenchyma (*Direct contact to left carotid artery, left optic nerve, frontal lobe parenchyma*) |

***Continued:* Supplementary table S2**

| Patient ID | PRT-planning images after surgery: Contrast enhanced MRI or CT | Indication criteria for PRT |
| --- | --- | --- |
| PA-15 |  | **Hardy**: IVE  **Knosp**: 4  **Criteria**:   - Size: Macroadenoma (*Max diameter > 10mm*) - Shape: Irregular (*Complex border configuration due to diffuse extrasellar extension reaching the inferior frontal lobe parenchyma. Delineable borders allowed target dose prescription to the GTV)* - Expansion: Extrasellar (*suprasellar, cavernous, sphenoid)* - Contact to OAR: Carotid artery, brain parenchyma (*Encasement of right carotid artery, direct contact to temporal lobe parenchyma*) |
| PA-16 |  | **Hardy**: IVE  **Knosp**: 4  **Criteria**:   - Size: Macroadenoma (*Max diameter > 10mm*) - Shape: Irregular (*Complex border configuration due to diffuse extrasellar extension reaching the right temporal lobe parenchyma, no MRI due to non-MRI-compatible pacemaker leads)* - Expansion: Extrasellar (*suprasellar, cavernous, sphenoid*) - Contact to OAR: Carotid artery, optic nerve, brain parenchyma (*Direct contact to carotid arteries, right optic nerve, right temporal lobe parenchyma*) |
| PA-17 |  | **Hardy**: IVE  **Knosp**: 1  **Criteria**:   - Size: Macroadenoma (*Max diameter > 10mm*) - Shape: Irregular (*Complex border configuration due to diffuse lining of the pituitary fossa and extrasellar extension reaching the left carotid artery. Delineable borders allowed target dose prescription to the GTV)* - Expansion: Extrasellar (*suprasellar, cavernous, sphenoid*) - Contact to OAR: Carotid artery (*Direct contact to left carotid artery*) |
| PA-18 |  | **Hardy**: IVE  **Knosp**: 4  **Criteria**:   - Size: Macroadenoma (*Max diameter > 10mm*) - Shape: Irregular (*Complex border configuration due to diffuse extrasellar extension reaching left temporal lobe parenchyma)* - Expansion: Extrasellar (*suprasellar, cavernous*) - Contact to OAR: Optic nerve, carotid artery, brain parenchyma (*Encasement of left carotid artery and left optic nerve, direct contact to left temporal lobe*) |
| PA-19 |  | **Hardy**: IVE  **Knosp**: 3B  **Criteria**:   - Size: Macroadenoma (*Max diameter > 10mm*) - Shape: Irregular (*Complex border configuration due to diffuse extrasellar extension reaching left temporal lobe)* - Expansion: Extrasellar (*suprasellar, cavernous*) - Contact to OAR: Carotid artery, brain parenchyma (*Encasement of left carotid artery, direct contact to left temporal lobe*) |
| PA-20 |  | **Hardy**: IVE  **Knosp**: 3B  **Criteria**:   - Size: Macroadenoma (*Max diameter > 10mm*) - Shape: Irregular (*Complex border configuration due to diffuse extrasellar extension reaching right temporal lobe)* - Expansion: Extrasellar (*suprasellar, cavernous, sphenoid*) - Contact to OAR: Carotid artery, brain parenchyma (*Direct contact to right carotid artery and right temporal lobe*) |
| PA-21 |  | **Hardy**: IVE  **Knosp**: 4  **Criteria**:   - Size: Macroadenoma (*Max diameter > 10mm*) - Shape: Irregular (*Complex border configuration due to diffuse extrasellar extension reaching right temporal lobe parenchyma. Delineable borders allowed target dose prescription to the GTV)* - Expansion: Extrasellar (*suprasellar, cavernous, sphenoid*) - Contact to OAR: Optic nerve, carotid artery, brain parenchyma (*Encasement of right carotid artery and right optic nerve, direct contact to right temporal lobe*) |

***Continued:* Supplementary table S2**

| Patient ID | PRT-planning images after surgery: Contrast enhanced MRI or CT | Indication criteria for PRT |
| --- | --- | --- |
| PA-22 |  | **Hardy**: IVE  **Knosp**: 4  **Criteria**:   - Size: Macroadenoma (*Max diameter > 10mm*) - Shape: Irregular (*Complex border configuration due to diffuse extrasellar extension invading left temporal lobe-, inferior frontal lobe parenchyma and reaching ventral brainstem)* - Expansion: Extrasellar (*suprasellar, cavernous*) - Contact to OAR: Optic nerve, carotid artery, brain parenchyma (*Encasement of right carotid artery and left optic nerve, direct contact to left carotid artery, left optic nerve, ventral brainstem invading into right temporal lobe and inferior frontal lobe*) |

*Abbreviations: CTV, Clinical target volume; GTV, Gross target volume; OAR, organs at risk*

Overview of the Hardy and Knosp classification systems for pituitary adenomas, describing sellar, suprasellar tumor extension and cavernous sinus invasion. Higher Hardy stages indicate increasing extrasellar extension, with Hardy stage IVE reflecting extensive extrasellar tumor growth, while higher Knosp grades indicate increasing cavernous sinus invasion. Knosp grade 1 indicates extension beyond the medial tangent of the intracavernous internal carotid artery but not beyond the intercarotid line; grade 2 indicates extension beyond the intercarotid line but not beyond the lateral tangent; grade 3 indicates extension lateral to the lateral tangent; and grade 4 corresponds to complete encasement of the intracavernous internal carotid artery. The above-mentioned criteria may be beneficial in guiding case-specific selection of proton radiotherapy.

**Supplementary Table 3:** Hormone control in functioning pituitary adenoma

| **Patient-ID** | **PA-Subtype** | **Hormone levels before last resection** | **Pre-PRT**  **hormone levels** | **Pre-PRT**  **hormone supplementation** | **Post-PRT**  **hormone levels**  **(last available follow-up)** | **Post-PRT**  **hormone**  **supplementation** | **Summary of endocrinologic report** |
| --- | --- | --- | --- | --- | --- | --- | --- |
| **PA-01** | GH | GH: 9.6 ng/ml  [Ref.: <3 ng/ml]  IGF: 745 ug/ml  [Ref.: 35-200 ng/ml] | GH: 8.4 ng/ml  [Ref.: <3 ng/ml]  IGF: 202 ug/ml  [Ref.: 35-200 ng/ml] | Hydrocortisone  [Dos.: 10mg bidaily] | GH: 3.0 ng/ml  [Ref.: <3 ng/ml]  IGF.: 190 ug/ml  [Ref.: 35-200 ng/ml] | Hydrocortisone  [Dos.: 10 mg bidaily] | Falling growth hormone levels after treatment. Inhibitory management with lanreotide not required. |
| **PA-09** | ACTH | Basal cortisol: 7.0 ug/dl  [Ref.: 5-23 ug/dl] | Basal cortisol: 7.6 ug/dl  [Ref.: 5-23 ug/dl]  SST (30min): 19.9 ug/dl  [Ref.: Cortisol Increase] | -/- | Basal ACTH (S): 12.3 pg/ml  [Ref.: <46 pg/ml]  Basal cortisol (S): 11.06 ug/dl  [Ref.: 5-23 ug/dl] | L-thyroxine  [Dos.: 36.5 ug daily]  Hydrocortisone  [Dos.: 10 mg bidaily]  Testosterone  [250 mg weekly] | Euthyroid state. Testosterone and cortisol within normal range. Hormone supplementation as indicated should be continued. |
| **PA-11** | GH | *Not available*  In referral report to the department of neurosurgery clinically stated as GH-secreting macroadenoma. | GH: 0.967 ug/l  [Ref.: < 10.00 ug/l]  IGF-1 70.50  [Ref.: 28-230 ng/ml] | Hydrocortisone  [Dos.: 10 mg daily]  L-thyroxine  [Dos.: 100 ug daily] | *Not available*  Endocrinologic follow-up documentation was available but did not include the results of hormone testing. | Hydrocortisone  [Dos.: 10 mg bidaily]  L-thyroxine  [Dos.: 50 ug daily] | Adequate hormone levels. Supplementation unchanged. |
| **PA-15** | P | *Not available*  In referral report to the department of neurosurgery clinically stated as prolactin-secreting macroadenoma. | Prolactin: 16.1 ug/l  [Ref.: 2.8-25.0 ug/l] | Hydrocortisone  [Dos.: 10 mg daily] | Prolactin: 10.2 ug/l  [Ref.: 2.8-25.0 ug/l] | -/- | No secondary pituitary insufficiency. No medication required |
| **PA-18** | GH | GH: 1.0 ng/ml  [Ref.: 0.05-1.23 ng/ml]  IGF-1: 356.4 ng/ml  [Ref.: 103-221 ng/ml] | GH: 0.5 ng/ml  [Ref.: 0.05-1.23 ng/ml]  IGF-1: 319.7 ng/ml  [Ref.: 103-221 ng/ml] | Hydrocortisone  [Dos.: 10 mg daily] | GH: 0.1 ng/ml  [Ref.: 0.05-1.23 ng/ml]  IGF-1: 83.8 ng/ml  [Ref.: 103-221 ng/ml] | Hydrocortisone  [Dos.: 10 mg daily] | Adequate hormone levels under glucocorticoid replacement. Low IGF-1 clinically non-significant, no supplementation required. |
| **PA-21** | GH | *Not available*  In referral report to the department of neurosurgery clinically stated as GH-secreting macroadenoma. | IGF-1 514 ug/l  [124-310 ug/l] | Hydrocortisone  [Dos.: 10 mg daily] | *Not available*  Endocrinologic follow-up documentation was available but did not include the results of hormone testing. | -/- | Adequate hormone levels. No supplementation required |

*Abbreviations: Ref., Reference range; Dos., Dosage; ACTH, Adrenocorticotropic hormone; GH, Growth hormone; S, Substituted; SST, Short synacthen test; P, Prolactin; IGF, Insulin-like growth factor.*

Shown data extracted from available referral- or endocrinologic reports.
